# Mobilizing community action to improve maternal health in a rural district in Tanzania: lessons learned from two years of community group activities

**DOI:** 10.1080/16549716.2019.1621590

**Published:** 2019-06-13

**Authors:** Andrea Solnes Miltenburg, Sandra van Pelt, Willemijn de Bruin, Laura Shields-Zeeman

**Affiliations:** a Institute of Health and Society, Section for International Health, Faculty of Medicine, University of Oslo, Oslo, Norway; b Department of Work and Social Psychology, Maastricht University, Maastricht, The Netherlands; c African Woman Foundation, Den Haag, The Netherlands

**Keywords:** Community participation, community group intervention, participatory learning and action cycle, reproductive health, maternal health

## Abstract

**Background**: Community participation can provide increased understanding and more effective implementation of strategies that seek to improve outcomes for women and newborns. There is limited knowledge on how participatory processes take place and how this affects the results of an intervention.

**Objective**: This paper presents the results of two years of implementing (2013–2015) community groups for maternal health care in Magu District, Tanzania.

**Method**: A total of 102 community groups were established, and 77 completed the four phases of the participatory learning and action cycle. The four phases included identification of problems during pregnancy and childbirth (phase 1), deciding on solutions and planning strategies (phase 2), implementation of strategies (phase 3) and evaluation of impact (phase 4). Community group meetings were facilitated by 15 trained facilitators and groups met monthly in their respective villages. Data was collected as an ongoing process from facilitator and meeting reports, through interviews with facilitators and local leaders and from focus group discussions with community group participants.

**Results**: The majority of groups prioritized problems related to the availability of and accessibility to health services. The most commonly actioned solution was the provision of health education to the community. Almost all groups (95%) experienced a positive impact on the community as results of their actions, including increased maternal health knowledge and positive behaviour changes among health care workers. Facilitators were positive about the community groups, stating that they were grateful for the gained knowledge on maternal health, and positively regarded the involvement of men in community groups, which are traditionally women-only.

**Conclusion**: The process of establishing and undertaking community groups in itself appeared to have a positive perceived impact on the community. However, sustained behaviour change, power dynamics and financial incentives need to be carefully considered during implementation and sustaining the community groups.

## Background

Over the past decades, the UN Millennium Development Goals (MDGs) have been successful in mobilizing public opinion, political leaders and international development agencies in the pursuit of human development and poverty alleviation [1–4]. More recently, the UN Sustainable Development Goals (SDGs) have been established as a follow-up to the MDGs. The SDGs give a central role to health, with attention for decreasing maternal and child mortality [5]. Most sub-Saharan African countries have not made sufficient progress in reducing the maternal mortality ratio [6]. Worldwide, maternal deaths have declined by 44% from 385 deaths to 216 deaths per 100,000 live births between 1990 and 2015 [7]. Despite progress, maternal death remains a problem in sub-Saharan Africa [6,8].

Causes of maternal death such as obstructed labour, obstetric haemorrhage, obstetric sepsis, hypertensive disorders of pregnancy and abortion are well known; however, there is no ‘magic bullet’ to prevent these deaths [9–11]. Improving maternal health demands implementation of a range of evidence-based strategies such as access to family planning, antenatal care, routine care during birth and emergency obstetric care [12–14]. The effectiveness of these strategies has been limited due to a disconnect between global policies and the local realities in which strategies are implemented [14]. This implementation gap is also evident in the lack of linkages between interventions in health facilities and the communities they serve [15,16].

Lack of understanding of the day to day realities that people face impedes the process of development and makes it difficult to translate the international development agenda into sustainable actions and change at the community level [3,4]. Community participation is used as a strategy to understand these local realities, especially in the field of health promotion, and to include local communities in planning and implementation of health programs [17–21]. Community participation can be defined as ‘*the collective involvement of local people in assessing their needs and organizing strategies to meet those needs*’, which increases the potential for ownership and sustainability [20,22–24]. Both active and passive approaches to community participation exist [20]. Where the passive approach is target-oriented and involves professionals who decide on objectives of health programs to which communities need to respond, the active approach empowers communities with the opportunity to be actively involved in decision-making on activities affecting their health [25,26].

Health programs or interventions for maternal and newborn health have traditionally been either passive (e.g. top-down, health service improvement) or active (e.g. bottom-up mobilization of communities to increase care seeking) in their approach [27,28]. Community participation provides opportunities for health program planners to introduce top-down ideas but execute them through a bottom-up approach [27,28]. Many community-based programs now comprise both service delivery and community participation components, which has shown promising results especially in the reduction of maternal and child mortality [25,29,30]. Several models and ranking of degrees of community participation between these two approaches have been developed and are based on a continuum of power-sharing (e.g. ranging from information sharing, mobilisation, collaboration and empowerment) [31]. Such a continuum implies the highest ranking of participation is the ultimate goal. However, it is increasingly acknowledged that different levels of participation can be considered appropriate at different times, depending on objectives, interests of the stakeholders involved and the political and social context. Additionally, beyond the goals of participation, the authors suggest that community participation can be seen as a way to develop individual and groups abilities to participate in processes of change [32].

Community mobilization through facilitated participatory learning and action cycles with women’s groups is one specific community-based program that developed in response to global realization that as well as strengthening services, maternal and newborn health strategies need to target capacity building of women, families and communities to ensure timely and appropriate care for pregnant women, mothers and newborns in the home-based settings [33]. Pathways of influence on critical outcomes, such as health behaviour, care seeking and health outcomes are complex. Current evidence on the positive effects of women’s groups was clearer for newborn mortality than the effect on maternal health and care-seeking outcomes. Partly, it is assumed this is due to the nature of the intervention including the heterogeneity of groups and activities. More broadly, however, the theory of change underlying the intervention is based on the belief that the participation process itself may encourage healthy behaviour, and lead to changes in social structures and social norms, which ultimately would encourage uptake of skilled care during pregnancy, birth and in the post-partum period [32]. Effects on health outcomes, including maternal morbidity and mortality, however, are in this respect contingent on simultaneous implementation of strategies to improve the available health services [33].

There is no universal blueprint for successful eliciting community participation through community interventions in low-resource settings. Most community-based health program reports in low-income countries omit details about how participatory processes took place and how this affected the results of an intervention. Participation itself can be seen as part of the intervention, and should be described and understood as such in order to relate this to the outcomes [31]. Participation can be viewed as a process along a continuum from information sharing, mobilisation, collaboration and empowerment [34]. Understanding the role of the participation process in relation to achieving health outcomes, for example, with regards to the successful women’s group intervention, requires disaggregation of the participation process [35]. This paper presents the results of two years of implementing community groups for maternal health care in a resource-scarce setting in Tanzania. We present socio-demographics of the community group members, their prioritised maternal health-related problems as well as their identified solutions and implemented interventions. In addition, we reflect on the implementation process, by analysing the context and conditions under which the intervention was implemented and how these might have influenced the outcomes.

## Methods

### Study setting

The community groups program, part of the Woman Centered Care Project (WCCP), led by the African Woman Foundation, was implemented in Magu District, Tanzania. Magu District is located in Mwanza Region, bordering Lake Victoria. The Lake Zone region is one of the poorest performing regions in terms of maternal and newborn health indicators [36]. The region is marked by a high total fertility rate of 6.4, a median birth interval of 29.6 months, a high unmet need for family planning (30%) and one of the country’s least performing regions in terms of facility births (50%) [36]. The Woman Centered Care Project (WCCP), led by the African Woman Foundation, started in 2013 and was officially launched in April 2014 with active participation from both community and district-level stakeholders. The project ended in August 2016. The WCCP’s primary objective was to improve maternal and neonatal health through a three-pronged intervention aiming to:

1. Increase health care seeking behaviour and demand for quality services targeting families and communities through community group activities;
2. Enable health facilities to provide appropriate, effective care by upgrading health facilities and providing training to health care workers;
3. Implementing a tablet-based Nurse Assistant App to enhance service delivery in rural dispensaries.

### Community group intervention

The community group intervention was conceptualised and developed as a result of exploratory research carried out in Magu District in 2012 and 2013, where both male and female community members proposed educational groups as a useful activity in their communities to increase knowledge on pregnancy-related issues. The community groups were inspired by the approach of women’s community groups carried out in India [37,38], Nepal [17], Bangladesh and Malawi [17,37–39], which resulted in substantial reductions in maternal and neonatal mortality rates as well as positive behavioural changes such as increased uptake of antenatal care services, institutional delivery, clean delivery practices for home deliveries and breastfeeding [17,37–39]. These women groups were assessed for impact and efficacy through a cluster-randomised controlled trial [17,37,38]. While randomised controlled trials constitute the gold standard [40], they are also resource-intensive. While working in the field, there is often a need to respond to immediate community needs and demands through implementation projects. The WCCP project therefore adapted the women’s community group model to suit the resource availability of the WCCP project as well as to the rural Tanzanian context [41]. This adaptation required a high degree of commitment from community and district leadership in order to generate buy-in for the community groups in the absence of financial resources.

In December 2013, local community members were selected according to criteria set by the project team (e.g. ability to read and write, being respected in their community) and trained to become facilitators for the community groups. Facilitators were selected from 7 of 8 wards (geographical sub-divisions) in the district. Wards were purposeful selected based on their geographic location in the district covering both areas close to Lake Victoria and wards situated more in-land, and based on accessibility (i.e. wards close to the main tarmac road for easier accessibility to reach villages). The facilitators were instructed to establish up to 8 groups in their respective villages. Prior to group establishment facilitators were trained to guide their groups to follow the Action Cycle, based on the participatory learning and action cycle [37–39], consisting of four phases over the course of two years. The four phases included identification of problems during pregnancy and childbirth (phase 1), deciding on solutions and planning strategies (phase 2), implementation of strategies (phase 3) and evaluation of impact (phase 4). Each phase consisted of approximately five meetings, depending on the needs of the groups. Information about detailed objectives for each meeting can be found in Additional file 1. Facilitators were trained prior to each phase and where necessary an extra training was scheduled. A training manual was developed by the project team, based on the Good Practice Guide of the Perinatal Care Project, Ekjut, University College London’s Centre for International Health and Development and Women and Children First UK [42]. Project team members provided the training in close collaboration with experts from the district council who provided short education sessions related to maternal and child health topics.

Facilitators were instructed to document group attendance, including demographic details, and activities. Supporting documents such as attendance forms, reporting formats and facilitation tools were developed using the good practice guide [41,42] and were adapted based on local needs and experiences of facilitators and project staff. Facilitators delivered reports of each group meeting to the project office in Magu Town on a monthly basis. During the training, activities of the previous meetings were evaluated and new meetings and tools discussed and prepared. Facilitators reported their findings as well as implementation challenges to the Ward District Council meetings, facilitated and attended by project staff, and which were held quarterly in each ward. Two staff members were appointed to supervise and guide the facilitators. They visited facilitators on a regular basis, had telephone contact where possible and observed almost each community groups at least once. The entire supervisory team consisted of six people. The team discussed progress and challenges in a bi-weekly meeting.

### Data collection process

Data used for this study consists of program data (quantitative and qualitative data), collected as an ongoing process of monitoring and evaluation of the WCCP community groups since their establishment (baseline data collection point) in February 2014 until the end-evaluation in November 2016. This includes both quantitative and qualitative information derived from facilitator and meeting reports (e.g. attendance lists, community meeting report), complemented with data derived from interviews with facilitators and focus group discussions (FGDs) with community group participants. Meeting reports and logs of facilitators were delivered in person at the end of each month and stored in archives in the office. Data of field visits, interviews and FGDs were digitalised in the same week of data collection and stored on the computer of the office.

The evaluation had four objectives: 1) to describe socio-demographic details of the facilitators and their groups, 2) to describe results of the intervention for all four phases of the action cycle 3) to describe the intervention implementation logistics and supervision 4) to understand how those involved with the intervention (local leaders, facilitators, group members) experienced the implementation process, including challenges and supportive factors for implementation. Table 1 outlines the indicators, data collection methods and data collection method for each objective. Interview and Focus Group Participants were purposefully selected based on their involvement with the intervention, and included all group facilitators, community leaders and group members who took part in community group activities.

**Table 1.** Process and outcome evaluation indicators and data collection methods.

Evaluation	Indicator	Data collection method	Data Source
Socio-demographic details of the community groups	Facilitators characteristics	Document review	Application and interview notes of 15 facilitators
	Community groups socio-demographics of participants	Document review	Attendance lists of all community groups
	Community groups establishment and continuation	Document review	Overview of active groups including number of meetings held.
Community group outcomes	Problem and strategies identification	Document review	Facilitator reports of 1922 group meetings
	Decision making process on problems and strategies	FGDs	Community groups (N = 9)
		Interviews	Facilitators (N = 18), group members (N = 21) and leaders (N = 3)
Logistics and supervision of the overall project	Local leadership involvement	Document review	Reports of 65 quarterly meetings
	Community meetings process	Document review	Facilitator reports of 1922 group meetings
		Observations	Research team reports of 51 community group meeting observations
	Facilitators development	Document review	Training reports of 4 one week trainings and 1 day workshop
		Document review	Reports of 16 supervision visits
	Project coordination	Document review	Reports of 66 project team meetings
		Interviews	Government officials (N = 3), project staff (N = 6), community members (N = 6)
	Evaluation of implementation costs	Document review and analysis	Quarterly and yearly financial reports
Experience and perceptions on the overall project	Leadership experience	Interviews	Local leaders (N = 11)
	Facilitator experience	Questionnaire Open ended (anonymous)	Facilitators (N = 15)
	Community group members experience	FGDs	Community Groups (N = 6)

### Data analysis

Quantitative program data was analysed in frequency tables in Microsoft Excel to count totals and percentages of community groups. Sociodemographic indicators among community group participants were counted and where continuous data was collected (age and number of children), mean and standard deviation were calculated.

All community group reports were translated from Kiswahili to English and entered into a database (Microsoft Excel). Interviews and FGDs were translated from Kiswahili to English, transcribed and also added to the database. All data was subsequently analysed based on the four objectives as presented above. Socio-demographic details of the groups were extracted from group meeting attendance lists (Evaluation objective 1). Outcomes of the community groups were analysed based on outcomes for each of the four phases as reported by the groups (Evaluation objective 2). Thematic coding was applied to identify and collate commonly discussed problems and solutions. A second level of analysis focused on deducing these themes into a cohesive final list of prioritised problems and related solutions, in which we distinguished the total number of times a certain topic was discussed during group meetings and the topics that were eventually prioritized by the groups. We also documented whether the prioritized solutions matched the identified problems. Outcomes of phase 3 and 4 from the community groups were assessed and collected from a number of sources, including meeting reports, observation reports, interviews and briefing meetings with facilitators.

A document analysis of the implementation process (Evaluation objective 3) entailed coding themes across interview transcripts, community meeting and team meeting reports, which were annotated and summarized and analysed according to process indicators as defined by Draper et al. (2010): leadership, planning and management, women’s involvement, resource mobilisation and monitoring and evaluation [31]. The indicators were scored in relation to the participation continuum: mobilization (1), collaboration (3), empowerment (5) and scores of 2 and 4 for intermediate types [31]. Definitions and values of the different levels of participation are provided in the supplementary material. Stakeholders’ experiences were assessed through thematic analysis of transcripts of interviews and FGDs (Evaluation objective 4).

## Results

### Characteristics of community groups and participant socio-demographics

In total, 15 community facilitators were trained through the program, of which 6 (40%) were men and 9 (60%) were women. The mean age among facilitators was 35 (SD: 7.33). In terms of education status, four facilitators had completed 11 years of education, and 12 facilitators had completed 7 years of education. All except one facilitator had children or became a parent during the project period. After the initial training of 15 community facilitators, 93 community groups were established in 2013 and 9 additional community groups were started later in 2014 and 2015. Facilitators had a 100% retention rate over the course of the project. Of the 102 community groups, 77 groups completed the four phases of the action cycle and were still active at the end of the WCCP (75% retention rate). Of the 25 community groups that were terminated, 9 groups dropped out in the first phase, 10 in the second phase and 6 in the third phase of the action cycle. The main reason for dropout was lack of financial support to run the groups, unmet expectations of group members and lack of personal incentives for facilitators and community group members. Other reasons for dropout included competing priorities over participating in the community group, decreasing group attendance and group motivation to continue. Table 2 presents an overview of the community group characteristics. The majority (n = 58, 57%) of the 102 community groups held at least 20 community group meetings, 19 groups (18.6%) held between 10 and 20 community group meetings, and 25 groups (24.5%) held 10 meetings or less. Community groups had on average of 23.8 group members. At baseline, the majority of community group participants were female (63.4% of the sample), married (78.9%), and have primary school as their highest level of educational attainment (66.3%). The mean age among participants was 38.4 (SD14.24) and participants had an average of 4.86 (SD 3.09) children. Table 3 presents the socio-demographics of the community group participants.

**Table 2.** Characteristics of community groups at baseline and end-line.

	Baseline (onset of community groups,	End-line (evaluation of community groups,
	2014)	2016)
**No of groups**		
Active groups	93	77
Additional groups	9	N/A
Terminated groups	N/A	25
Number of Registered members in community groups	2071	1365
Average no of members per group	23.8	18.2

N/A: not applicable

**Table 3.** Socio-demographics of community group participants at baseline and end-line.

Variable	Baseline (onset of community groups) 2014	End-line evaluation of community groups) 2016
**Total number of participants**	2071	1365
**Gender**		
Female	1313 (63.4%)	929 (68.1%)
Male	714 (34.5%)	435 (31.9%)
Unknown	44 (2.1%)	1 (0.1%)
**Marital status**		
Single	223 (10.8%)	110 (8.1%)
Married	1633 (78.9%)	1094 (80.1%)
Widow	119 (5.7%)	111 (8.1)
Divorced	8 (0.4%)	10 (0.7%)
Unknown	88 (4.2%)	40 (2.9%)
**Level of education**		
None	330 (15.9%)	160 (11.7%)
Primary (not completed)	140 (6.8%)	65 (4.8%)
Primary (complete)	1374 (66.3%)	1038 (76.0%)
Secondary school	114 (5.5%)	51 (3.7%)
Higher education	3 (0.1%)	1 (0.1%)
Unknown	110 (5.3%)	50 (3.7)
**Age**		
**Mean (SD)**	38.4(14.24)	39.75(12.64)
<20 years	61 (2.9%)	26 (1.9%)
20–34	846 (40.8%)	475 (34.8%)
35–49	700 (33.8%)	532 (39.0%)
50–65	332 (16%)	234 (17.1%)
>65 years	118 (5.7%)	61 (4.5%)
Unknown	14 (0.7%)	37 (2.7%)
**No of children**		
**Mean (SD)**	4.86(3.09)	4.95(2.92)
None	140 (6.8%)	72 (5.3%)
1 or 2	383 (18.5%)	220 (16.1%)
3 or 4	443 (21.4%)	333 (24.4%)
5 or 6	488 (23.6%)	327 (24.0%)
7 or 8	332 (16.0%)	220 (16.1%)
9 or more	249 (12.0%)	160 (11.7%)
Unknown	36 (1.7)	33 (2.4%)

### Community group outcomes

Of the 77 groups that completed the four phases of the action cycle, the problem discussed most frequently across groups was ‘disrespectful behaviour of health care workers’ (discussed in 80.5% of the groups). This was followed by problems categorized as ‘lack of services at the health facility’, (76.6%), ‘lack of health education’ (64.9%), ‘general poverty’ (61.0%), ‘lack of health workers (59.7%), ‘medical problems related to pregnancy’ (58,4%) and ‘poor health-seeking behaviour and/or birth preparedness of women’ (53.2%). The majority of groups prioritized problems related to the availability of and accessibility to health services. Table 4 presents the outcomes for each of the phases including prioritized problems, prioritized solutions, actions taken and evaluation.

**Table 4.** Prioritized problems, solutions and actions taken by the community groups*.

	Problem (Phase 1)	No of groups (%)	Solution (Phase 2)	No of groups (%)	Actions (Phase 3)	No of groups (%)	Evaluation (Phase 4)	No of groups (%)
1	Health services too far	19 (25)	Providing health education	29 (38)	Providing health education to individuals/families, at health facility or during meetings	63 (82)	Behavioural change women/husbands (e.g. women are more prepared, more ANC visits)	47 (61)
2	Lack of service in health facility (e.g. equipment, medication)	15 (19)	Construct health facility	19 (25)	Setting up a community health fund	33 (43)	Increase of knowledge (of women, men, community)	44 (57)
3	Lack of health workers at the health facility	14 (18)	Increase health seeking behaviour for ANC	19 (25)	Collaborating with the government (inform leaders, have meetings, write letters, ask for help)	21 (27)	Behavioural change health workers (e.g. less corruption, better behaviour, transfer of HCW)	18 (23)
4	Medical problems related to pregnancy (e.g. bleeding, convulsions)	11 (14)	Promote facility birth	17 (22)	Preparing and taking action for construction work (e.g. collecting stones, water, cement)	18 (23)	Health outcomes improved (e.g. less maternal deaths, less still births)	17 (22)
5	Lack of health worker housing	10 (13)	Promote health insurance	9 (12)	Raising awareness on disrespectful behaviour of health providers	11 (14)	Good response from or collaboration with the community	17 (22)
6	Disrespectful behaviour of health providers	10 (13)	Construct health care worker housing	9 (12)	Encouraging community member to join the health insurance	9 (12)	Services improved (e.g. more equipment, supplies, shorter waiting time)	14 (18)
7	Lack of health education (e.g. poor knowledge or awareness)	8 (10)	Renovate existing health facility	4 (5)	Generating income for the community (chicken program/honey business)	7 (9)		

* Some groups had indicated more than 1 prioritized problem or solution so the total percentage can be more than 100%. Not all groups prioritized problems aligned with prioritised solutions. Rows are ordered based on percentages of groups and therefore do not always relate to one topic. No = Number. HCW = Health care worker. ANC = Antenatal care.

For 60 of the 77 groups (78%) the prioritized solutions aligned with the prioritized problems, even though groups had different solutions for the same problem. As an example, solutions aligning to the problem of ‘*health facilities being too far*’ could be providing ‘health education’ on birth preparedness to women, establishing a ‘community health fund’ with pooled funds for emergency transport or groups deciding to ‘build a new health facility’ in their own village. Even though groups proposed a variety of solutions to their problems, the majority of the groups decided to initiate action related to providing health education to the community (82%). This decision was sometimes initiated in addition to other solutions and sometimes as a replacement of their initially identified solutions as the initial solutions were deemed to be too complex to implement. In phase 4, groups evaluated their actions and reported on the groups’ perception of the outcome of their activities. Most groups (73 of the 77 groups, 95%) believed that some of their other actions they had taken as a facilitator (e.g. provide health education) had some positive impact on the community even though their chosen solution in the community group may not have been completed. The majority of the groups (78%) reported a positive change in the behaviour of women and husbands including increased knowledge on issues concerning maternal health. Thirty-two groups (42%) reported that they were satisfied with their achievements as a group. Stated achievements include positive behaviour change among health care workers, improved health outcomes, and improved health care services such as increased availability of instruments available after community groups were implemented and more explanations given by HCWs to patients who were community members from that particular village.

Community group decision-making processes were based on the steps provided in the action cycle, usually through a voting system in the groups whereby each member had one vote. Groups prioritized problems and solutions that they expected to make the biggest change in their lives or targeted the most important problem(s). Although poor behaviour of health care workers was a frequently discussed topic in all the groups, often with heated debates and use of anecdotes, groups expressed the following reasons not to prioritize this issue: either because they lacked inspiration for solutions, they thought of it as an easy problem which did not require the projects support, they did not believe the community was capable to change this or they expected to address it along with the other, more pertinent, problems. During supervisory visits and facilitator feedback meetings, it appeared that some of the groups took actions to address this problem. Actions varied from reporting the issue of problematic behaviour among health care workers to local leaders to ensure that health care workers would receive a warning, arranging village meetings to request health care workers to change their behaviour, educating the community on health facility rules, encouraging women to prepare better for delivery, and encouraging women to ensure good personal hygiene and bring sufficient supplies to reduce negative encounters with health care workers. However, few groups documented these actions. Additionally, groups frequently sympathised with the working conditions of health care workers and therefore thought that improving their work conditions would improve health workers’ job-satisfaction and subsequently their behaviour towards patients. Actions included: attempting to increase the number of health care workers, building a dispensary closer to the village and ensuring accommodation for health care workers near the dispensary.

### Implementation logistics and supervision

The project implementation strategy included careful consideration of several important intervention components including appropriate male and female involvement in the community participation process, eliciting different levels of community participation and leadership, and evaluation strategies.

A key initial priority of the WCCP intervention was the involvement of women in the intervention itself. Male involvement was also considered important and was specifically elicited in this project and is often neglected in other community-based maternal health projects. This led to the project decision (supported by community members and local leadership) to have both male and female group facilitators.

**Figure 1.**
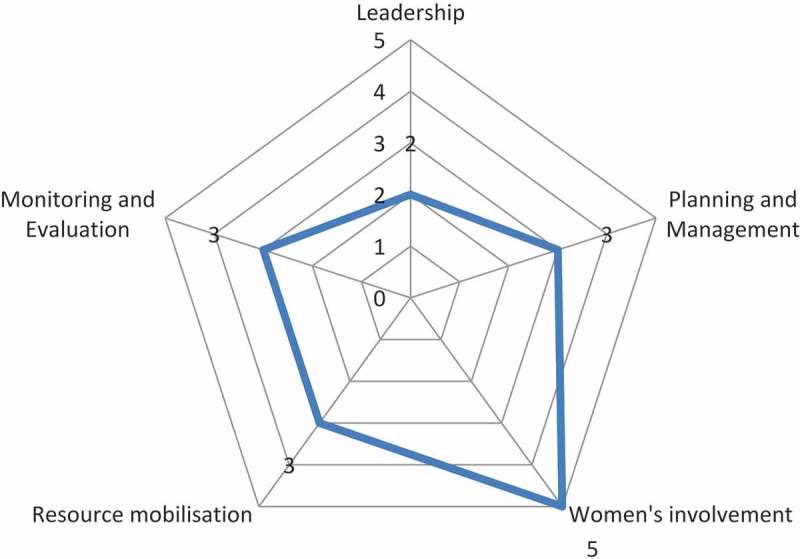
Community group intervention participation scores according to dimensions in Draper et al.’s process evaluation framework [31].

Another factor affecting the implementation process was the turnover of the project team and local leadership. Throughout the two-year implementation period, the project team went through several leadership and staff member changes, which led to new introductions of staff to the communities, which came with different communication styles between the project team and community members. Additionally, national and local elections took place during the implementation of the community groups, resulting in the change of government leadership at crucial times during the project, delaying implementation and shifting facilitator priorities. Table 5 presents the value given across the continuum of community participation which is depicted in Figure 1.

**Table 5.** Process evaluation of implementation process based on Draper et al. (2010) [31].

Indicators	Evaluation
Leadership	Project initiated by project staff members in collaboration with community leadership and in response to findings of the needs assessment. Leadership structure formed through a top down approach based on local protocol. At district level a steering committee was formed including representatives of the district council, ward leadership and project staff. At ward level the ward district council was responsible to oversee the project implementation. Village/hamlet leaders were asked to provide support. Facilitators played an important role in voicing the concerns of community members.
Planning and Management	Project staff informed the community how to participate and decided the projects focus (on maternal health), goals and activities (community groups). Community invited to participate within a predetermined structure, but free in how and what they wanted to do. Activities reflect community priorities and involved local people including existing groups. Through training of facilitators some transfer of skills occurred.
Women’s involvement	The active participation of both men and women in positions of decision-making and responsibility was a main objective. Maternal health projects are often mainly focused on women involvement, active involvement of men was a conscious decision. At the same time, the project ensured facilitators and leaders (where possible) were female. Also the project staff had both male and female members.
Resource mobilisation	The majority of funding for the intervention was from outside the community. The initial intention was to get financial buy-in from the community leadership, but this never materialized. Communities offered non-financial resources including time, space and local expertise. Professionals allocated the use of resources, although this was done in consultation with the steering committee. Facilitators played an active role in development of their materials and how the coordination of the project should be structured.
Monitoring and evaluation	Project staff design evaluation approach and performed analyses, but facilitators and their groups were involved in data collection. A broad definition of ‘success’ was used, based on the groups own perspectives of their achievements. Responses to monitoring findings are jointly decided and community feedback is both sought and given.

To ensure that local leadership was continuously engaged and mobilized during the course of the community group development, implementation and evaluation, the project developed a participation plan. In some aspects, the leadership (e.g. district medical officer, village and ward leaders) was supportive in taking actions at the community level in maternal health. As an example, local ward authorities allowed facilitators to report their progress on a quarterly basis during the Ward District Committee meetings (WDC meetings). This action was primarily meant to ensure that facilitators would be assisted with any logistical challenges faced while doing their work and to ensure some ownership of the interventions by the ward committees. The interaction and dialogue during the WDC meetings had several unexpected benefits. First, Ward District Committee meetings started to occur on a more consistent basis; prior to the project, a lack of governmental (financial) support meant that WDC meetings happened irregularly. As the project provided a small stipend for the meetings to be held, committee meetings occurred on a more regular basis, which provided a unique platform in bringing stakeholders together. In addition to discussing project activities, the community groups also provided a platform where local matters could be addressed collectively (e.g. how to fix a leaking roof of a health facility or how to come to terms with the shortage of water in the village). Second, in addition to reporting logistical challenges, community facilitators would inform the committee members of the problems discussed within their groups. Many of these problems were unknown to the leadership; in particular, they were often not aware of the many challenges at the health facilities.

Through the community group activities, community members were able to decide for themselves what actions should be taken to address their identified problems within their local communities. The groups met monthly, and it took about a year to implement their solutions identified in the community groups. For some of the facilitators, it was difficult to maintain group interests, as some individuals wanted to validate their own priorities and concerns, and there were changes in community member composition throughout the year. During interviews, facilitators reported that they relied heavily on project team visits in order for the community to believe their activities were part of a larger project and to validate the performed work themselves. Supervision visits by the project staff were initially limited due to insufficient project funds to cover these expenses, and it took several months until all facilitators had been visited. Community expectations that were beyond what the project could deliver, mistrust towards the facilitator and lack of tangible benefits were major challenges faced by the facilitators for which they needed project team members to support their efforts. Facilitator training mostly addressed facilitation skills and implementation of the action cycle, as well as ensuring motivation and capacity building of the facilitators.

The total costs of the intervention were approximately 28.000 USD, including the cost of implementation as well as operational and intervention development costs. Expenses related to project staff and administration costs accounted for roughly one-third of the total expenses and were calculated in percentage of their contribution to this specific implementation. The project team attempted to generate buy-in from the village and district government in terms of payment of facilitator salaries, bicycles or other contributions to the project in the form of cost sharing. Despite several promises by district leadership to contribute, this never materialized. In terms of monitoring and evaluation, the project relied heavily on facilitator reports. Facilitators had to bring their reports to the project team every month, which also gave opportunities for the team to meet with the facilitators. It was not always clear to facilitators which aspects of group meetings to report, even though reporting skills were part of the training. Continuous supportive supervision and observations of group meetings by the project team helped to increase mutual understanding of the group processes and facilitator influence.

### Stakeholder experience of the intervention

All facilitators (n = 15) reported that they have enjoyed their role as a facilitator throughout the project. They expressed satisfaction with obtaining skills to be a community facilitator and receiving education and information about maternity care. On a more personal level, facilitators expressed in the interviews that they appreciated that they could use their role as a facilitator to contribute to the community, and expand their network within the community. All facilitators mentioned the positive effect their facilitating work had on their family in terms of providing a source of income, an audience to share their new knowledge, improvement of social skills, and increased confidence. Most of the facilitators expressed that they, together with their family, became local advisors on maternal health issues which increased their social value in their community:

*‘Through this work I gained a social position which encourages me to be a facilitator; I got knowledge and strength in community meetings.’* (Facilitator quote of questionnaire)

Challenges were expressed as unrealistic expectations of the community group members to the facilitators or were related to logistics such as distance between the villages where community meetings were held. All facilitators expressed that community group members continued to ask a small financial compensation to attend the community group meetings, which was not provided within the scope of the project, which subsequently led to changes in attendance rates of community members. Most of the facilitators explained that group members expected to receive education within the groups and considered them more as teachers, not understanding the role of a facilitator. Some facilitators complained about the reliability of local leaders, transport problems, and insufficient salary.

One unique feature of the community groups in Magu district is the inclusion of men in the community groups. This was received positively by the facilitators and community members, as it was repeatedly mentioned in the interviews that the inclusion of the men was valuable:

*‘Men also are parents, and fathers, so due to that it is very important for them to be also within the group, everything that a mother or a woman knows also a man or a father should know, that is why they are needed in the group.’* (Female community group member during interview)

Community group members reported during the focus group discussions that the project did not provide direct individual benefit. During all the focus group discussions, group members reported they were unable to achieve their goals due to lack of financial support by the project. Some community group members mentioned that they did not experience any tangible changes as a result of their participation in the groups. They related specifically to their remaining inferior status with lack of involvement or participation at the local leadership level. The most substantial benefit of the project, reported during the focus group discussions, seemed to be the platform for community support and dialogue that the community group offered. Community group members acknowledged that it makes a difference now that they are together as a group and local leaders respond to that:

*‘Last time one of my neighbour his daughter was pregnant, when she attended ANC (antenatal care) no service was given to her so she went back home and explained to her daddy that she didn’t get any services because she didn’t went with her husband, so my neighbour came to my home place and explained that to me and I was ready to explain to her what is supposed to happen according to the lesson I learned from our facilitator and I advised him that he will need to go with her daughter next time and he went on behalf of her husband and finally she was provided with antenatal care.’* (Community member during FGDs)

Participation in community groups among local leaders and decision makers varied considerably. Some leaders, primarily village or sub-district leaders, were active group members, facilitated group formations and emphasized key messages of the community groups in other community meetings. Others were aware of the project but were to a lesser extent involved. Leaders indicated that they valued the roles of the facilitators. One leader mentioned that some facilitators were mobilized to become ‘change agents’ which would be of benefit for the community in the long run.

*‘I would like to provide special thanks to the facilitators for their spirit on educating the community about maternal health. (…) Facilitators are patient and they mobilized which facilitates community members.’* (Ward executive officer during interview)

One village leader expressed the project did not have much benefit as he perceived the project was not reaching many people and was unable to address major health facility challenges. One of the leaders expressed concern regarding the project activities, timeline of involvement and sustainability.

*‘I was thinking about this project to be sustainable due to the low progress of the outcomes. (…) The project activities which are conducted in the community can’t bring outcome within a short time it needs more time to see the big impact*.’ (Ward executive officer during interview)

## Discussion and conclusion

The findings presented in this paper highlight the benefits and challenges, as well as the complexity of implementing a community group intervention in a low-resource setting with limited funds. The community group intervention implemented in Magu District, Tanzania as part of the Woman Centered Care Project intended to encourage local communities to identify problems related to maternal health and identify solutions for these problems. The evaluation of the project outcomes and processes reveal that the process of setting up and forming community groups in itself can have a positive effect on the community, beyond the initial goals of the action cycle and goals set by the groups. The platform created an opportunity for group members and other community members to share challenges related to pregnancy and childbirth and ask for help. Additionally, it facilitated a communication line between community members and various community leaders, which otherwise did not exist or was not operational. One key factor that is suggested to determine successful health programme interventions through community participation is related to the extent to which involved stakeholders are strengthened in their capacity [43] to ‘define, assess, analyse and act on health (or any other) concerns of importance to their members’ [44]. Focussing on capacity strengthening of individuals and communities could function as a step towards achieving program goals. This includes, to enhance their ability to develop new knowledge, skills and attitudes, which are required to function and collaborate to reach a certain goal. Evaluating community capacity can function both as a means and end allowing projects to scrutinise achievements beyond the impact on health outcomes alone [45]. The level of participation in different program elements directly influences to what extent the capacity of communities and its individual members is likely to be strengthened. Based on our process evaluation, we believe there are three aspects that require careful consideration when implementing community participation interventions for maternal health. These include the need for a transformation of attitudes and behaviour, reflection on who ultimately holds power and control, and the role of financial incentives to achieve community group sustainability.

Engaging in health promotion projects through community participation requires a transformation of attitudes and behaviour beyond focussing on primarily the programs goals (e.g. increased health care seeking behaviour). It requires a change in the attitude of individual community members, towards having a mindset and ability to critically assess the underlying causes of inequalities which foster poor maternal health and to feel capable to address these more structural issues [46,47]. Working across the four phases of the action cycle requires specific skills, such as report writing, evaluation and reflection. The complexity of such skills for rural communities is often underestimated [48]. Underlying causes of socio-economic inequalities span beyond health (e.g. poverty, gender inequality), and unfortunately addressing these inequalities was not within the project goals. Many of the groups in this study made decisions and chose strategies that were not realistic or achievable in the project time period and thus required project staff intervention (e.g. deciding on unrealistic solutions, lack of connection between problems and related solutions, strong influence of facilitator and local leadership in group focus for problem identification). At times, solutions (strategies) selected by community groups were not motivated by an individual or community-level decisions or ownership over their identified problems, but rather motivated by an expectation that the project staff or local government would respond favourably to the strategy with more support and/or funding. This may have influenced how community members perceive their actual role of participation and ultimately what level of participation is reached, regardless of the project objectives [49,50]. To our knowledge, no other studies promoting community groups or women’s groups report similar situations. A systematic review by George et al. (2015), on community participation in health research, identified very few studies engaged communities in identifying or framing the problems to be addressed. Additionally, the authors highlight that levels of community participation are influenced by a continuous changing balance of power within projects, both between participants, project staff, and with the external health system and political decision makers [51].

Power dynamics in local communities can greatly influence how different stakeholders participate. Community members, both men and women may fear expressing their opinions, in particular with regards to health facility issues, out of fear for not being assisted at these same facilities when the time comes. Comparable hesitance to voice concerns and expectations about health services was observed through a situational analysis on social accountability for maternal health services in the Democratic Republic of Congo [52,53]. Facilitators functioned as a sort of intermediary between individual community members and local leadership. This allowed for expressing community concerns without this leading back to individuals or specific groups. Ways in which participation is structured can sometimes work around such power dynamics, creating ways of communication that are perceived safe by all those involved. Few studies evaluating community participation reflect on the programmes and staff changing roles and positions in relation to the community. Similar to most intervention projects in low-resource settings, our involvement in the community did not extend beyond three years due to financial constraints. Although phasing out of a research location is recommended [54], rather than stopping activities from one moment to the next, few projects allocate resources to such a sustainability component of the program [55]. Ultimately, power and control of project resources and decisions in terms of intervention focus and coordination and management of the overall project remain limited to project staff from the beginning until the end. Similar to findings of a systematic review on community participation in health system research, themes to target and design of our intervention was determined outside of the community [51]. Although the decision to include community participation as part of the project’s objective was based on needs assessments performed, working with community groups was more heavily based on results from a review of the literature [33,38,39,56–59] and less on the involvement of relevant stakeholders.

Community participation is a dynamic process. Regardless of the purpose or intention of eliciting community participation, it cannot be forced, and external decisions to perform interventions relying on community participation require substantial investment and time to build local trust and commitment to obtain community engagement, and eventual ownership over the intervention or programme [27]. In particular, for research projects it is also important to build research capacity, this has not been given sufficient priority and our project contributes to maintaining the existing inequality with authors of this paper also being based in high-income countries [51]. Our findings show that external groups (e.g. foreign NGOs) can play a role in laying the foundation for participation to be possible, which does not necessarily require a large influx of funds to stimulate this process. Nevertheless, all stakeholders involved in our project commented on the lack of financial contributions or incentive to participate, which may have contributed to some groups discontinuing and reduces the likelihood of sustaining groups after ending the project. The impact of incentives on sustaining a community group in a resource-poor setting cannot be underestimated [60], and could therefore be considered as a limitation of our research. However, the increase in funding and financial incentives would be unlikely to have resulted in sustained commitment as soon as the funds subside. It is promising that an increasing number of publications present the positive impact of community participation activities on health outcomes for disadvantaged populations [61], which has also resulted in community participation strategies being included at policy level [62]. However, sustainability of community participation is only likely if it is sufficiently integrated in existing structures and appreciated by official institutions and its representatives [63]. Given the relatively low investment required in human resources for deploying an intervention like this for the benefits it brings, a community group intervention may be interesting for the local policymaker. Community group approaches leverage the insights of existing community members rather than relying on the health care workforce, and limited operational costs outside of costs to run groups and monitor and evaluate their implementation make such an approach attractive. That being said, the case for investing in community mobilization groups as a method for improving maternal health has to be weighed against other competing policy priorities in a given region or country. Further research is needed to determine how this can be achieved and how participation processes can be sustainable without external mobilization and funding.

The strength of our study is our evaluation of the participation process, which to the best of our knowledge, has not been studied or included extensively in the evaluation process in other studies employing elements of community participation [51,64]. A limitation of the study is the fact that we evaluated program data and qualitative data, as it was not possible to evaluate the effectiveness of the intervention on outcomes in a controlled trial design. Furthermore, implementation and evaluation processes were not completely distinct, as researchers involved in the evaluation were also involved in the implementation. Although this allows for a clear understanding of all the documents and sheds light on important components and perspectives of the implementation process, it carries a risk of bias towards the interpretation of the data. Nevertheless, we believe the analysis performed presents a realistic representation of the implementation process, achievements and challenges faced in the field. Our study was not designed to determine whether and to what extent the intervention contributed to improved health outcomes, which is a limitation. Outcome evaluation relied on facilitator reports of their groups’ qualitative evaluation processes. It is very likely the groups were biased to report positive outcomes, and we cannot confirm if their perceptions were valid. It is also very likely facilitators had a major steering influence on the choices made by community groups related to their priority problems and solutions. Nevertheless, with the detailed report of our evaluation, we believe in the further development of successful community initiatives to target health issues in low research settings.

## Paper context

Community participation, and the Action Cycle in specific, has proven to be a successful tool to understand communities and to implement strategies to improve maternal health. Little knowledge exists on the relation between the implementation process and the results of the intervention. In this paper, results of the implementation of the Action Cycle are presented together with a reflection on the implementation process itself and contextual factors and how this affected the results.
